# Postpartum psychosis treatment: review of mother-baby units

**DOI:** 10.1192/j.eurpsy.2022.2227

**Published:** 2022-09-01

**Authors:** M.L. Costa, A. Elduayen Vila

**Affiliations:** 1 Hospital Universitario Severo Ochoa, Psychiatry, Leganes, Spain; 2 Universidad Alfonso X el Sabio, Medicina, Madrid, Spain

**Keywords:** Perinatal psychiatric disorders, Epidemiology, PSYCHOTIC DISORDERS, mental health policies

## Abstract

**Introduction:**

Postpartum Psychosis is an underdiagnosticated psychiatric condition that may be suffered by mothers within a year since delivery. It is a severe syndrome in which symptoms such as delusions, hallucinations and disorganized thinking may appear. The traditional approach of admitting the mothers separated from their children has shown harmful consequences. This has led to the creation of Mother-Baby Units (MBU), psychiatric admission units dedicated to full-time housing mothers and their babies.

**Objectives:**

To evaluate the evidence available regarding the potential benefits of MBU not only for the mothers, the babies, but for the mother-baby bond. To analyse postpartum psychosis risk factors and prognosis.

**Methods:**

A thorough review of scientific literature and databases regarding postpartum psychosis and MBU has been carried out. Additionally, international mental health care guidelines for perinatal mental disorders were analysed.

**Results:**

A wide range of related aspects were studied for the present work, including characteristics of the patients, differences in the self- assessment scales of depression, anxiety, postpartum attachment of the mother to the baby at admission and at discharge and the work and social adaptation. Other studies analysed the percentage of mother-baby separation at discharge, as well as the most frequent delusions, and the potential effect of childhood trauma on these patients.

**Conclusions:**

The available evidence suggest that MBU may be helpful for the improvement of the mental health in women suffering perinatal mental disorders and for the building of a secure attachment style in the baby. The results of the interventions included in MBU programs also show promotion of a positive mother-baby relationship.

**Disclosure:**

No significant relationships.

